# Latest‐Late Fertility? Decline and Resurgence of Late Parenthood Across the Low‐Fertility Countries

**DOI:** 10.1111/padr.12334

**Published:** 2020-04-27

**Authors:** Eva Beaujouan

**Keywords:** late fertility, age at birth, fertility postponement, men and women, Europe, low fertility countries

## Abstract

After decades of fertility postponement, we investigate recent changes in late parenthood across low‐fertility countries in the light of observations from the past. We use long series of age‐specific fertility rates from the Human Fertility Database (1950–2016) for women, and new data covering the period 1990–2016 for men. In 1950, the contribution of births at age 40 and over to female fertility rates ranged from 2.5 to 9 percent, but then fell sharply until the 1980s. From the 1990s, however, the prevalence of late first births increased rapidly, especially so in countries where it was initially lowest. This has produced a late fertility rebound in the last two decades, occurring much faster for women than for men. Comparisons between recent and past extremely late (age 48+) fertility levels confirm that people are now challenging the natural fertility barriers, particularly for a first child.

## Introduction

Age at childbearing continues to rise across the low‐fertility countries. In 2016, mean age at first birth was often reaching 28–30 years for women, with Italy having the highest at 31 years (Sobotka et al. 2018). In the early 1970s, first births occurred before age 25 on average in all European countries and the United States, so this increase is considerable (Neels et al. 2017). Biological factors are never, and cultural factors not always, favorable to childbearing at older ages. Rising levels of fertility at less fertile ages go hand in hand with increasing number of unsuccessful attempts to have children at late ages: Research has drawn attention to the psychological distress experienced by men and women who do not succeed in having children they desire, particularly a first child (McQuillan et al. 2003; Wischmann and Thorn 2013). Nonetheless, increasing shares of women are having children in their 40s and beyond (Prioux 2005; Billari et al. 2007a; Beaujouan and Sobotka 2019). It is thus important to establish a complete picture of late fertility trends in the low‐fertility countries, and of its variations across different contexts, to allow researchers to reflect on this change in a long‐term perspective and to better assess its extent and potential repercussions.

In the early 1950s in Europe, the United States, and Japan, large families were very common (Sardon 1992; Van Bavel et al. 2018). At that time, “late” parenthood was by no means unusual, consisting mostly of high order births (Prioux 2005; Beaujouan and Sobotka 2019). The years of rapid economic development after World War II were an exceptional period in the modern history of the family (Coontz 2006; Hareven 2010). A growing share of the population adopted early marriage, with women's age at first marriage falling to a record low in the 1950s in the United States and Japan and in the 1970s in Europe (Sardon 1992). Adherence to family norms gradually strengthened, with two‐child families becoming very common (Brzozowska, Beaujouan, and Zeman 2017) and late fertility more exceptional (Billari et al. 2007a; Beaujouan and Sobotka 2019).

The change in individual values from the early 1970s, brought about by the Second Demographic Transition (SDT), women's increased labor market participation, their rising levels of education, and the availability of effective contraception led to a general postponement of family formation and first births (van de Kaa 1987; Nicoletti and Tanturri 2008; Lesthaeghe 2010; Ní Bhrolcháin and Beaujouan 2012; Neels et al. 2017). With the rise in economic uncertainty in the last quarter of the twentieth century, it also took longer for couples to achieve sufficient financial stability to raise a family (Kreyenfeld, Andersson, and Pailhé 2012). In today's society, it is now socially acceptable and, often, economically necessary, to start a family much later than in the 1970s, and this has resulted in a sharp rise in late first and second births, and in overall late fertility over recent decades (see, e.g., Billari et al. 2007a).

The age at which one becomes a “late” mother or father cannot be defined without reference to a period and a country (Moguérou et al. 2011). The definition of late childbearing is subjective and is embedded in the fertility levels and norms prevailing across time and space. However, from age 40, childbearing events become rarer among women, making that age a good threshold for empirical study (Billari et al. 2007a; Beaujouan and Sobotka 2019); women's age‐related physiological inability to have children increases exponentially from age 35, and by age 40 more than one third of women are generally estimated to be permanently sterile (Leridon 2008). Across all European countries in 2006, women above 40 were commonly perceived to be too old to consider having any more children (Billari et al. 2011). Age 48, for its part, is a good threshold for extremely late childbearing among women, as natural births beyond that age are exceptionally rare (te Velde and Pearson 2002; Leridon 2008). In Sweden, for example, first birth rates at that age did not rise to a visible level until the late 1990s (Billari et al. 2007a, Figure 3). With the use of assisted reproductive technologies (ART), conditional birth rates at age 48 and above increase visibly beyond the “chance occurrences” observed in natural conception (Billari et al. 2007a, 163–64). The age dynamics of childbearing are very different for men, and a biological threshold for late fertility is not as easily defined as for women. They are able to have children much later and are less constrained by social norms on age limits (La Rochebrochard et al. 2006; Sartorius and Nieschlag 2010; Billari et al. 2011). A parallel exploration of female and male ages at “late” fertility would provide a useful means to refine the definition of late parenthood for men.

Trends in late and extremely late fertility have rarely been examined in a comparative perspective over the very long term. In particular, we know very little about the timing and scale of these trends after the onset of fertility postponement, and whether they varied across countries. Moreover, existing studies rarely focus on men, or do so only for specific countries (Bessin, Levilain and Régnier‐Loilier 2005; Prioux 2005; Moguérou et al. 2011). Using age‐specific fertility rates (ASFRs) from the Human Fertility Database and Human Fertility Collection, we compare 19 European countries, the United States, and Japan. Focusing first on women, we study the very long‐term prevalence of “late” (40–59 years of age) and “extremely late” (48–59 years) childbearing in several low‐fertility countries between 1950 and 2016. We compare the speed of diffusion of late first births across low‐fertility countries since the onset of postponement and study whether the increase is still ongoing several decades later. We then contrast age limits for male and female late fertility over time. In the final discussion, we suggest new avenues for research on important issues related to postponed childbearing and late fertility.

## Contrasting patterns of late childbearing since 1950

Late fertility is most prevalent both when large families are frequent because high‐order births occur at higher ages, and when family formation is postponed to late ages because late first and second births are more numerous (Prioux 2005; Billari et al. 2007a; Moguérou et al. 2011). Family size in the 1950s and the speed of its decrease until the 1970s, as well as the timeframe of delayed family formation in the following years, are decisive factors for understanding cross‐country variations in late fertility. We describe here these changes across time and place and detail the underlying mechanisms that may have been driving late childbearing since the onset of “fertility postponement.”

### Decline in prevalence of large families and in late parenthood until the 1970s

Contraception and marriage timing were important mechanisms behind family size and age at childbearing after World War II. In the 1950s, most births took place within marriage, and age at marriage was quite high, with variability across Europe (Sardon 1992): 24–25 years in the West and around 23 in Poland and Hungary. After marriage, as contraception was only partially effective, individuals were at risk of having children even at late ages, so late parenthood was frequent (Prioux 2005; Billari et al. 2007a; Beaujouan and Sobotka 2019). Across countries for which the relevant data are available, the share of very large families varied widely, but could be considerable: more than 30 percent of all women had four or more children in the Netherlands and Portugal, around 20 percent in Hungary, the United States, and Italy, but just 15 percent in the Czech Republic (calculations from Human Fertility Database). In 1950, total fertility rates (TFR) ranged from 2.0 to 3.5 children per woman, with the lowest fertility levels observed in the north of Europe (2–2.5), and the highest levels in the non‐European English‐speaking countries and Japan (3–3.5) (Sardon 2006; Frejka, Jones and Sardon 2010). In the Central and Eastern European (CEE) countries, TFRs ranged from 2.5 to 3.5 children per woman (Sardon 2006; Sobotka 2011). We expect countries with many large families in the 1950s (for instance, the Netherlands and Portugal) to have had the largest shares of late parents, all birth orders combined. They certainly also experienced extremely late (age 48+) high‐order births. We postulate that the countries of Central and Eastern Europe, given their earlier family formation and relatively low fertility had a rather low prevalence of late fertility in the 1950s.

The two following decades were a time of increasing conformity of family behaviors (Coontz 2006). With the advent of effective contraceptive methods in the early 1960s, very large families soon became less frequent in all these countries, replaced by the new norm of the two‐child family (The ESHRE CapriWorkshop Group 2010; Mills et al. 2011; Brzozowska et al. 2017). In Europe, mean age at first marriage reached a low of 22–23 years around 1975, with little age variability (Sardon 1992). Particularly, late marriage retreated. For instance, in Austria, female marriage rates after age 40 were 3.5 times lower in 1980 than in 1950 (Prioux 1992). The United States was a major exception. Age at marriage in that country had already reached its lowest level—around 20 years on average—in the 1950s, and remained below 21 until the early 1970s (Schoen and Canudas‐Romo 2005). In Japan, mean age at marriage was already on the rise in the 1970s, after a low of around 23 years in 1947 (Population Statistics of Japan 2017). On average, first births were earliest in the 1970s (Neels et al. 2017). Under the combined effect of early family formation and shrinking numbers of large families, late fertility reached its lowest level in the 1970s (Prioux 2005; Billari et al. 2007a; Beaujouan and Sobotka 2019). We posit that this decrease was universal, though earlier in Japan and starting from lower levels in Eastern Europe and that this conformity of family behaviors led to more uniform contribution of late fertility to the overall fertility levels. We also postulate that extremely late childbearing almost disappeared at that time.

### A phase of expansion of late parenthood driven by later entry into parenthood

The latest and still ongoing phase, more generally known as the SDT (see, e.g., Lesthaeghe 2010), is a period of profound societal transformation toward diversity in the family (van de Kaa 1987; Inglehart and Baker 2000; Bonvalet, Clément and Ogg 2014). In the presence of efficient contraception and in the midst of growing individualism, childbearing lost some of its “centrality,” and became mostly a way to improve one's own life satisfaction and self‐fulfillment rather than to comply with the norms of familism (van de Kaa 1987). This shift in norms and values went together with a change in family‐related behaviors: later union formation, spread of nonmarital cohabitation and childbearing, growing union instability, and rising voluntary childlessness (Sobotka 2008; Sobotka and Toulemon 2008; Coleman 2013; Vergauwen 2016). Men and women started to retreat from childbearing at younger ages, and then—generally but not always—went on to have children at later ages (Ní Bhrolcháin and Toulemon 2005; Beaujouan and Toulemon 2019).

Age at first birth rose sharply, starting in the early 1970s in Western Europe and in the early 1990s in the East (Sobotka 2004; Neels et al. 2017). Only in the United States was the rise less dramatic: In 2014 mean age at first birth was still 26.3 years, against around 29 in many other countries (Mathews and Hamilton 2016; Neels et al. 2017). The later start in the east did not prevent countries such as Hungary from reaching a mean age at first birth of 28 years in the early 2010s, just below most of Western Europe. In parallel, fertility rates decreased until the early 2000s, reaching the lowest levels in the southern European countries, CEE countries, and Japan, but remaining relatively high in the English‐speaking, Nordic and Western European countries (Frejka et al. 2010; Sobotka et al. 2018). Since 2010, the rates have started to converge at between 1.5 and 1.8 children per woman across the European regions and the United States in 2016, except in southern Europe and in Japan, where they remain extremely low (at around 1.3 and 1.4 children per woman, respectively).

Retreat from early childbearing (say, before 30) has been attributed to a wealth of causes partly embedded in the SDT, such as efficient contraception; rising preference for competing activities such as leisure or work; greater union instability; worsening economic security, etc. (Mills et al. 2011; Barclay and Myrskylä 2016; Beaujouan and Toulemon 2019). Additional factors have led to a mechanical deferral of childbearing by a few years. These include the longer time spent in education and the rise of youth unemployment, which delay economic independence (Winkler‐Dworak and Toulemon 2007; Kreyenfeld et al. 2012; Ní Bhrolcháin and Beaujouan 2012; Neels 2015). A second set of factors affect fertility in the second part of the life course (Beaujouan and Toulemon 2019). The large increase in healthy life expectancy has made it feasible to envisage parenthood at older ages, and improvements in perinatal healthcare have reduced the risks associated with later childbearing (Prioux 2004; Kotelchuck 2007). New opportunities to have children at later ages have also emerged, such as late union formation and repartnering after union dissolution (e.g., stepfamilies). Finally, in the countries under study, between 20 and 40 percent of the women in the 1966–1970 birth cohorts were university graduates, against 5–20 percent in the 1936–1940 cohorts (Brzozowska et al. 2017). Given that highly educated women have their children particularly late (Rendall et al. 2010), this has certainly been a driver of late childbearing over the last decades. The rise in late and very late fertility may have been particularly strong in settings where unions are now most diverse and highly educated women are most numerous, for instance, in the Nordic countries.

New dynamics have arisen from these new situations: at more advanced stages of their life course, adults have become used to living a “child‐free” life, have built a career and developed other life goals, so their attitudes to childbearing are liable to change (see, e.g., Keizer, Dykstra, and Jansen 2008; Buhr and Huinink 2017; Gemmill 2018). While today more than 90 percent of childless women in their early 20s still wish to have a child, as they advance in life the intentions of those who are still childless tend to change (temporarily or permanently), particularly after age 30 (Gray, Evans, and Reimondos 2013; Rybińska and Morgan 2019). These mechanisms used to result in generally low childbearing intentions at age 40. On the other hand, the enduring “preference for children” across the low‐fertility countries means that a nonnegligible number of women still wish a child even after reaching older ages: with fertility postponement, the proportion of childless women at age 40 wishing to have a child has increased in the last decades, from 3.5 percent in 1986 to 29.8 percent in 2016 in Austria, for example (Beaujouan 2018). This indicates a change in attitudes toward late childbearing, but particularly reflects the fact that constraints at earlier ages are so strong that people now want children at ages where they are much less likely to succeed. Postponement of childbearing to successively older ages may be particularly frequent in countries where the employment conditions at young ages are more difficult, and where conflicting demands, such as the difficulty of combining work and family life, are particularly salient (Kohler, Billari, and Ortega 2002). Southern Europe is affected by a combination of these two factors (Zuanna 2001; Caltabiano 2016), while the impact of the latter constraint is extreme in south‐east Asia (see, e.g., Frejka et al. 2010; Gauthier 2016). These countries, where TFRs are the lowest and childlessness highest, may therefore exhibit a particularly large share of late and extremely late births, with a shift toward “latest‐late” fertility.

The rise in late childbearing across the low‐fertility countries is interesting to study because it is not only the inexorable consequence of birth postponement, but also certainly reflects the diversity of childbearing norms and constraints across different countries. After the low levels of the 1970s, we expect to see a rise in the prevalence of late first births across all countries following the onset of postponement. As this trend started later in Eastern Europe, we postulate that late fertility in the east has not (yet) reached the levels observed in the other countries. However, the rise may have been faster as these Eastern countries lived through uncertain economic times and their family‐friendly policies were discontinued in the 1990s as policies became increasingly oriented toward the market (Sobotka 2011): Their age at first birth has increased more rapidly than in most countries of Western Europe. In addition, we expect the prevalence of late first childbirth to have increased the most in countries with severe obstacles to earlier childbearing, particularly in southern Europe and Japan. Finally, the extent of fertility postponement has increased the number of people trying to have a child at unusual ages for a first birth (Beaujouan 2018). Combined with the increasing availability of ART, this increases the number of people potentially able to have a child at these ages. Extremely late first births may thus become more visible in the very recent period.

## Women and men under different constraints

Men and women differ strikingly in the amount of physiological time they have to form and enlarge a family (Fisch and Braun 2005). This difference is of major importance in societies where family formation is becoming increasingly delayed: while men can adapt to postponement, the problem is more complex for women, whose chances of a conception leading to a live birth decrease substantially from age 35. Researchers have used microsimulation to show that for women, fertility postponement leads to more involuntary childlessness and smaller families, less so in the Czech Republic and Austria, but more so in Spain and the Netherlands (Leridon and Slama 2008; te Velde et al. 2012; Habbema et al. 2015). Demand for ART developed in recent decades becomes particularly high from age 35. In 2015 in the United States, around 1 percent of all births before age 35 were achieved using in vitro fertilization, versus 3.8 percent at aged 35–37 and 37.7 percent after age 44 (Beaujouan and Sobotka 2019). However, ART cannot yet be used to reverse age‐related biological limitations (Leridon 2004). From age 35, any additional delay comes with an increased risk for women of not having the birth they desire, be it a first or higher order child.

Male infertility also increases with age, but later and much less drastically than among women (La Rochebrochard et al. 2006; Eisenberg and Meldrum 2017). This biological difference is socially reinforced (Schmidt et al. 2012): men are often several years older than their female partners (Ní Bhrolcháin and Sigle‐Rushton 2005) and social norms on the upper age limit for having a child are stronger for women than for men (Billari et al. 2011). In the 2006–2007 European Social Survey covering 25 countries, the average social cutoff point for childbearing was 41.7 years for women, but much higher, at 47.3 years, for men (Billari et al. 2011). Under these constraints, men and women have unequal access to parenthood at later ages, although the extent of this difference remains to be assessed (but see Moguérou et al. 2011 for France). In Western and Northern Europe in particular, women's and men's educational and employment trajectories are becoming increasingly similar, as are their family trajectories (Lesnard et al. 2016). While women are having their children later and later, is this also the case among men?

Research that systematically compares male and female family behavior, or addresses only men's behaviors, has become more frequent but is largely outweighed by the numerous studies focusing on women (Keizer, Dykstra, and Poortman 2010; Lappegård, Rønsen, and Skrede 2011; Moguérou et al. 2011). Childbearing and family health have long been considered as female matters, and data on men have rarely been collected. Moreover, the quality of available data on male family events is recognized to be of poorer quality than that of those available concerning women. For example, men may not always be aware of their parenthood in the case of extrapartnership births; more often than for women, men are not at home when a survey is conducted and another person present in the household (“proxy”) answers the questions on their behalf; and men may not report their children as accurately as women (Rendall et al. 1999; Joyner et al. 2012). This is compounded by the difficulty of identifying men's children; while women are directly linked to their child in the birth registers, the father's characteristics are often missing (Dudel and Klüsener 2018). Male age‐ ASFRs, necessary to our study, are no exception, and are generally not routinely available (Schoumaker 2015). We take advantage of a series of male ASFRs recently constructed by Dudel and Klüsener (2018) to compare male and female late fertility.

## Data and method

For women, our study relies on birth indicators available from the Human Fertility Database (Jasilioniene et al. 2007), which gives data on numbers of births—overall or by birth order, by year or by birth cohort—drawn from birth registers and other official and validated sources. The female population exposure is estimated using data on population size and deaths from the Human Mortality Database. For our study, we use female period ASFRs, age‐specific first birth rates (ASFR1), and age‐specific second birth rates (ASFR2) available for women aged 15–59 in most countries. In a few countries, they are available only up to age 55, but given that births at age 55–59 represent less than 0.1 percent of births at age 40–59 this will not influence our study of late parenthood. Before being entered into the database, the original data are not always provided by single year of age, so “round” ages (e.g., 40 or 45) are used to present late fertility. For a few countries, we do know fertility rates by single ages until age 49 (and then in a broad 50–59 age group, except in Hungary and Spain, 50–55); we use these countries for our study of extremely late fertility.

The ASFR series generally cover the years from the 1950s, spanning back to the first half of the twentieth century in a few countries. Age‐specific rates by birth order are not available in all the countries where ASFRs are available, and the series are generally shorter. For the study of long‐term trends among women, we thus focus on six countries for which data on first births span back to the early 1950s (Austria, Hungary, Italy, the Netherlands, the United States, and Japan since 1967), and that are also distributed across the main low‐fertility areas: respectively, the German‐speaking countries, Central Europe, Southern Europe, Western Europe, and the English‐speaking countries. Japan is free standing because low fertility there preceded the other East Asian countries by 20 years (Frejka et al. 2010). Since we also have data by single year of age for these countries, we can present them consistently in the parts of the paper covering late childbearing trends among women. Note that the female indicators we constructed for this study are provided in the online Supporting Information for all the countries and all available years, even when the country was not selected in the paper. For Austria we use data from the Human Fertility Collection (Šťastná and Sobotka 2009), and for Italy data provided directly by IStat (“mothers’ ASFRs”).

We adopted Sobotka's definition (2004, 52) for the year of onset of postponement, that is the first year after 1965 when mean age at first birth showed an increase which lasted for three or more calendar years and led to a total increase of at least 0.5 years of age. He proposes an alternative definition to that of Kohler et al. (2002), for whom the year of onset of postponement is the first in a span of three years during which the mean age at first birth increases by more than 0.3 years. After checks for two thirds of the countries using the Human Fertility Database series of age at first birth, we concluded that the onset calculated using Sobotka's definition was generally two to three years earlier than that obtained under the alternative definition, but appeared as the beginning of a series rather than one (sometimes isolated) point, and better represented slow starts (as in the exceptional cases of Hungary where it was 13 years earlier than under Kohler et al.’s definition, and Japan, where it was 17 years earlier). Except in these two countries, the choice of definition does not greatly affect the relative timing of onset of postponement between countries nor the strength of the increase in late fertility levels because these levels were rising very slowly at the beginning of the postponement phase.

Male ASFRs since 1990 are provided by Dudel and Klüsener (2019) who reconstructed them based on birth registers, and more precisely on the age of the fathers of children born each year up to age 59. They are available in the Human Fertility Collection. The proportion of missing values in these data is variable, however, ranging from small (less than 2 percent, e.g., in Sweden) to considerable (up to 47 percent on one year in Denmark), though it is generally below 10 percent (Dudel and Klüsener 2019). To address this problem, they tested a conditional method for attributing missing paternal birth dates that also relies on the known mother's age, comparing it to the usual attribution along the fathers’ age distribution. They found a maximum bias of around 1 percent in mean age at childbirth when using their method (Dudel and Klüsener 2018, supplementary material), that still depends on the proportion of missing values and on their distribution. Unfortunately, countries for which male data are available do not always correspond to the countries for which we have long female series, so the countries for which male and female ASFRs are compared differ somewhat from those analyzed in other parts of the paper.

We first calculate the contribution of births at age 40 and above to the TFR by summing the ASFRs at age 40–59 and dividing them by the TFR. We calculate the contribution of first births at age 40 and above to the first birth rate (TFR1) in the same way. This is done for all years across all countries. In addition, we present the sum of first, second, and all birth rates at age 48 and above in the countries where this is possible (the choice of age thresholds is explained in the introduction). To examine more closely the speed of diffusion of late births across different countries, we represent the relative increase in the contribution of first births at age 40 and above to the TFR1, taking as starting point the year of onset of postponement. Last, to compare men and women, we show the contribution to the TFR of all births at aged 40+/45+ for women, and at aged 45+/55+ for men. Contributions are shown for two time points (1990 and 2014), and we calculate the change over time separately for men and women to see whether the recent increase in the share of late births is stronger among men or women.

## Results

### Late childbearing trends among women

**FIGURE 1:**
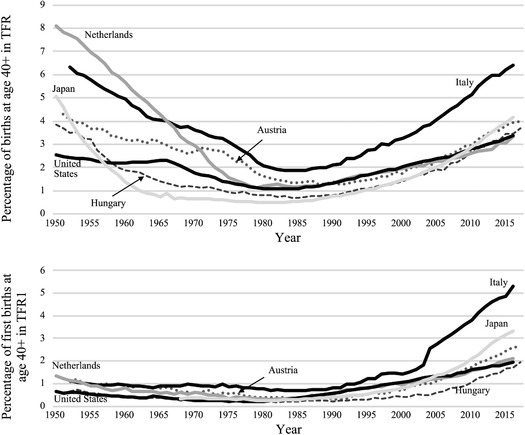
Contribution of women aged 40–59 to (a) total fertility rate, (b) first birth rate, 1950–2015, selected countries NOTE: Trends for all countries are available in the Human Fertility Database and trends in contribution to second birth rates are provided in the online Supporting Information. SOURCE: Human Fertility database, Human Fertility Collection for first births in Austria until 1983 and Italy until 2003.

Figure 1 shows late fertility trends since the 1950s in selected countries across the main low‐fertility regions. We first give the contribution of births to women aged 40 and above to the TFR (Figure 1a). In the 1950s, this contribution was higher than today and was notably much more diverse across countries—it generally corresponded to the share of very large families at that time (see online Supporting Information for more countries such as Portugal). It then declined until 1985 in most low‐fertility countries and increased again afterwards, to levels which are generally more similar today than in the 1950s. In the Netherlands and Japan, the initial drop was very sharp due to an extremely rapid decrease in family size. Late fertility remained most frequent in Italy (and more generally Southern Europe, see online Supporting Information) after the initial decrease. In countries like Austria and the United States, the drop was moderate, notably following a resurgence of larger families during the baby boom. Finally, in Hungary—as in the other countries of Central and Eastern Europe for which data are available—the share of late fertility was among the lowest, notably due to the low prevalence of large families since World War II.

As noted before, family size limitation is a factor behind the initial decrease in late fertility, but it does not explain its subsequent resurgence. Fertility postponement, on the other hand, has increased the share of first and second births occurring after age 40. Taking the extreme example of the Netherlands, while 90 percent of births at age 40 and above in the 1950s were third or higher order births, today, 60 percent are first and second births (author's calculations from Human Fertility Database). The contribution of first births at age 40 and above to first birth rates started increasing substantially from the mid‐1980s onwards, ranging between 2 and 5 percent in the mid‐2010s in the countries under study (Figure 1b). Late first births were slightly more numerous in the 1950s than in the deeper low of the 1980s, because marriages were taking place later at that time, spreading to later ages, and contraception was not yet very effective: There were more persons “at risk” of having a late first child.

Second births should also be mentioned here. The ideal number of children is predominantly two in Europe (Sobotka and Beaujouan 2014), and most people want a second child, particularly in Western Europe. Second births occur later than first births on average, so given their high frequency, they are likely to occur more frequently at late ages than first births. The country patterns of the contributions of late second births to second birth rates are relatively similar to those for first births, so they are not shown here, but they are available in the online Supporting Information. More second than first births are late, and this has always been the case. On the other hand, they are also more likely to be constrained by age‐related infertility than first births. Second births at later ages may thus be increasingly limited by the age barrier, and their proportion among all second births, if not stagnant, may rise less quickly than in the past. Such an effect is not generally observed, however. The contribution of second births at age 40 and above to the second birth rate has increased strongly since the late 1980s; only in Italy, where that contribution is particularly large, has the increase slowed down somewhat in recent years.

In Table 1, we examine births and first births at age 48 and above, events which are so rare that the rates are expressed in units per 10,000 women. Mean age at first birth, and first and second birth rates at age 40 and above are given for reference here and throughout the paper.

**TABLE 1:** Key indicators of very late first births in selected low‐fertility countries

			TFR per 10,000 women	First birth rate per 10,000 women		Second birth rate per 10,000 women	
Country	Year	Mean age at first birth	Age 48–59	Age 40–59	Age 48–59	Age 40–59	Age 48–59
Austria	1955	25.0	5.5	85.3	0.5	146.0	0.6
	1975	23.6	2.1	42.4	0.4	64.7	0.7
	1995	25.7	0.8	32.9	0.0	42.9	0.2
	2015	29.2	5.3	178.5	2.5	183.7	1.6
Denmark	1955	‐	2.8	‐	‐	‐	‐
	1975	23.9	0.7	22.0	0.1	30.8	0.0
	1995	27.2	0.2	45.0	0.0	76.1	0.1
	2015	29.2	3.7	140.4	1.0	197.6	0.7
Hungary	1955	23.4	5.6	61.6	1.0	97.0	0.5
	1975	22.6	0.9	21.3	0.2	33.8	0.2
	1995	23.8	1.1	19.2	0.2	29.3	0.2
	2015	27.9	1.9	116.5	1.0	147.4	0.4
Italy	1955	25.8	7.6	81.8	0.2	119.4	0.3
	1975	24.7	3.8	79.8	0.2	115.8	0.2
	1995	28.1	0.8	73.0	0.3	105.4	0.5
	2015	30.8	13.3	318.7	9.3	353.0	4.9
Japan	1955	‐	7.3	‐	‐	‐	‐
	1975	25.7	0.3	27.0	0.1	27.9	0.0
	1995	27.8	0.2	36.0	0.0	44.2	0.0
	2015	30.0	1.5	222.4	1.0	211.2	0.3
Netherlands	1955	26.2	9.9	79.6	0.2	134.5	0.5
	1975	25.2	2.2	24.2	0.2	43.2	0.3
	1995	28.4	3.1	54.5	0.8	73.5	0.3
	2015	29.7	4.2	152.1	2.1	168.5	0.9
Norway	1955	‐	‐	‐	‐	‐	‐
	1975	23.5	0.5	25.7	0.3	52.5	0.0
	1995	26.3	0.4	44.7	0.1	81.2	0.0
	*2014*	28.7	7.4	140.3	1.2	205.3	0.8
Spain	1955	‐	‐	‐	‐	‐	‐
	1975	25.1	16.3	90.5	1.9	126.1	1.0
	*1996*	28.4	1.3	59.8	0.5	74.2	0.3
	2015	30.7	10.1	335.6	5.8	345.9	2.9
Sweden	1955	‐	3.7	‐	‐	‐	‐
	1975	24.3	1.0	27.2	0.1	38.2	0.0
	1995	27.2	1.0	63.8	0.3	100.8	0.2
	2015	29.2	10.1	189.6	2.6	250.2	2.0
United States	*1935*	23.4	13.2	30.4	0.4	41.6	0.3
	1955	22.8	4.7	54.5	0.4	87.1	0.2
	1975	22.8	0.8	15.5	0.0	22.4	0.0
	1995	24.6	1.3	70.1	0.3	87.8	0.3
	2015	27.0	8.3	133.3	2.8	165.6	2.1

NOTE: ‐ = not available. TFR at age 48–59 corresponds to the sum of ASFR from age 48 to 59; likewise for the other indicators. In Hungary and Spain only, the last age is 55. In the United States for the years 1975 and 1995, we cover only births up to age 50. First and second birth rates at age 48–59 are smoothed over the two surrounding years (e.g., 2014–2016 for 2015). For Spain, in 1955, fertility after age 45 is not available by age but by age groups in the original data used for the construction of the Human Fertility Database rates, so we cannot show it; there is also a problem in the classification by birth order between 1980 and 1995, so we show 1996 here. Sums of ASFR, ASFR1, and ASFR2 at aged 48–59 for all the countries available in the Human Fertility Database are provided in the online Supporting Information.

SOURCE: Human Fertility Database, Human Fertility Collection for Austria, IStat for Italy.

Fertility rates summed over age 48 and above were often higher than now or at equivalent levels in the 1950s (Table 1): more women were having many children so more were giving birth at these ages. Births at age 48 and above are thus not only the result of increased use of ART; they were already taking place in earlier times when exposure was high, that is when married women were at risk of conception because of ineffective contraception. First and second births at age 48 are becoming more visible, however, and are more frequent today than at any other observed time. While mostly between 0 and 0.5 children per 10,000 women in 1995, first birth rates summed for age 48 and above now often reach more than 2 per 10,000 women. In Italy and Spain, they reached, respectively, 9.3 and 5.8 children per 10,000 women in 2015. The numbers remain minute, but the increases are very large. Note that conditional first birth rates (i.e., only among women who are still childless) at age 48 also rose between 1995 and 2015, but we could not retrieve this information for all countries or for all years, so it is not shown. This reflects an increased propensity to have children among childless women at age 48 and above and suggests that the increasing demand for extremely late births observed in the last two decades has been at least partly met thanks to ART. Interestingly, Billari et al. (2007a, Figure 3) observed very little increase in first births after age 48 in Sweden because their series ended in 2002, but first births at those ages started reaching more substantial numbers shortly after (see also online Supporting Information).

The sum of second birth rates at age 40 and over remains larger than that of first birth rates. However, second birth rates at age 48 and above are generally lower today than first birth rates at those ages. This was not always the case in the past, and we observe the opposite in Austria, Italy, and the Netherlands in 1955. This reflects a change over time in the dynamic of first and second births; in a regime of natural conception, the share of late second births was mechanically larger than that of late first births. Today, a large proportion of extremely late births are achieved via assisted reproduction, at least in the United States (Beaujouan and Sobotka 2019), and our observations suggest that having a child at very advanced reproductive ages reflects a desire to have “at least one child” rather than to enlarge a family.

## Fertility postponement and late motherhood

**FIGURE 2:**
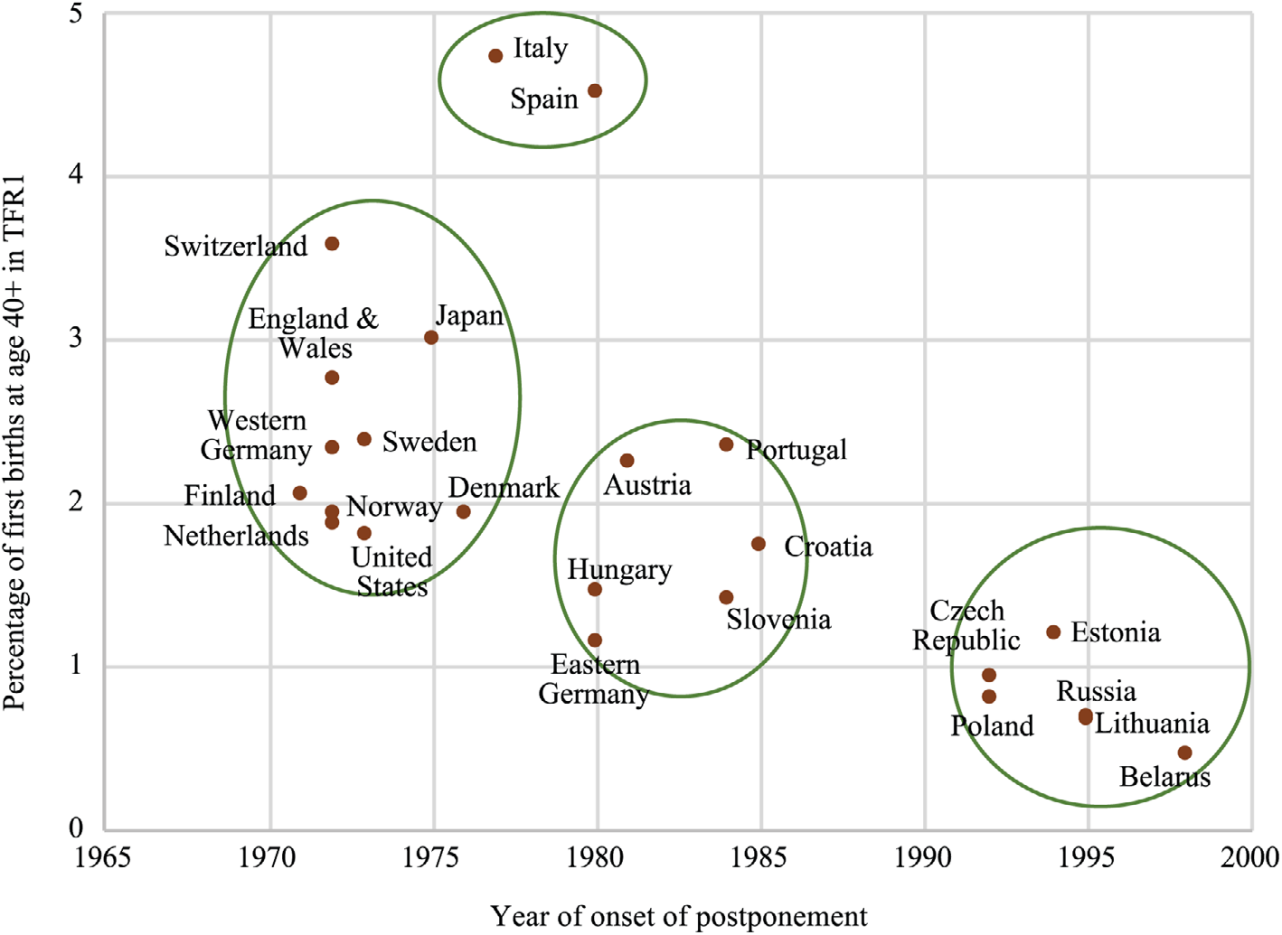
Contribution of women aged 40+ to first birth rates, 2014 versus year of onset of postponement, low‐fertility countries NOTE: Year 2013 for Western and Eastern Germany. The year of onset of postponement is the first year after 1965 when MAFB showed an increase which lasted for three or more calendar years and led to a total increase of at least 0.5 years of age (Sobotka 2004, 52). SOURCES: Human Fertility Database and Collection, and Sobotka (2004, table 3.3, 53) for the year of onset of postponement.

Analysis of trends in late first births since the onset of fertility postponement reinforces our cross‐country comparison. Year of onset of postponement and contribution of late first births to current first birth rates are correlated across countries (Figure 2). Where postponement started earlier—in the early 1970s primarily in Western Europe, Japan, and the United States—the contribution of first births after age 40 to the total first birth rate in 2014 was largest (3–5 percent after age 40, Figure 2). Where postponement started in the first half of the 1980s, 2–4 percent of first births were to women aged 40 or higher, except in the South. Finally, in Eastern Europe, where mean age at first birth started increasing in the 1990s, late first births are in the lowest range of 2–3 percent.

Independently from their year of onset of postponement, shares of late first births have increased spectacularly in Spain and Italy, accounting for around 6 percent of the total first birth rate. Though late parenthood was always prevalent in these countries where familism seems to contribute to later home leaving and thus to later family formation (Reher 1998; Zuanna 2001), the recent levels possibly reflect the increased constraints to family formation resulting from the lasting economic crisis. Interestingly, in the countries considered as most advanced in the SDT and with the largest share of highly educated women, such as the Nordic countries, there is not a particularly strong propensity to have children at age 40 or above.

The upturn in late first births since the onset of postponement also helps in differentiating countries. Some countries where the contribution of late first births to total first birth rates was originally very low, as well as some countries where postponement started later, may be catching up.

**FIGURE 3:**
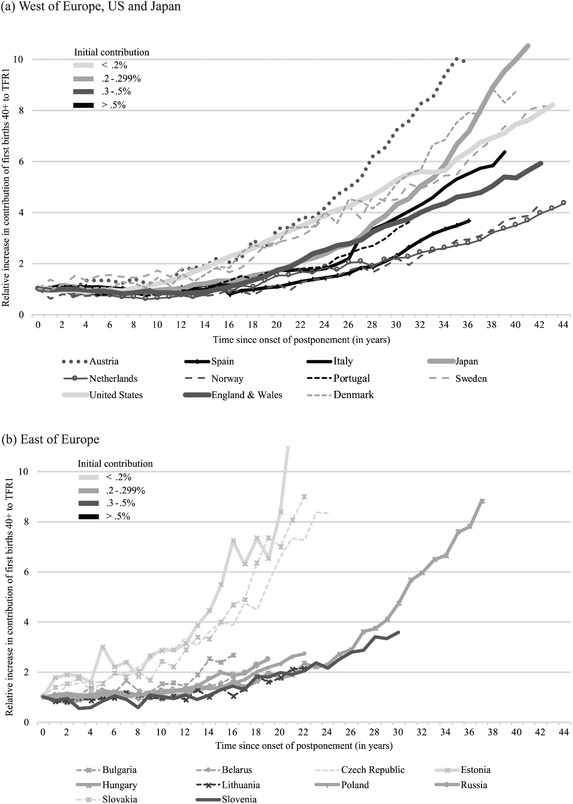
Increase in contribution of first births at 40+ to first birth rates in (a) West of Europe, United States and Japan and (b) East of Europe; starting point = year of onset of postponement. Detailed by level of contribution at onset of postponement SOURCES: Human Fertility Database and Collection and Sobotka (2004, Table 3.3, 53) for the year of onset of postponement.

Figure 3 displays the change in the contribution of first births at age 40+ to total first birth rates, relative to the level on the year of onset of postponement. The gray scale indicates the level of contribution on the year of onset: the darker it is, the larger the share represented by first births at age 40+ in the total first birth rate. The West and East of Europe are on two separate graphs to recall their very different years of postponement onset, but their gray scale is the same, giving a continuity to the comparison. The diffusion of late first births was slow and rather similar across most countries for the first 15–20 years after the onset of postponement. Only in countries with initially low levels of late fertility within their group did diffusion occur faster (United States and Sweden in the West) or even much faster (Czech Republic, Slovakia, and Estonia in the East). Austria was an exception; despite an initial contribution of 0.37 percent to first births, late first births spread very fast and intensively. After 20 years, the speed of diffusion began to differentiate across countries. In Japan, for example, 39 years after the onset of postponement the contribution of births at age 40+ to the first birth rate had increased almost 10‐fold. In other countries such as the Netherlands or Norway, the increase was only fourfold over 42 years. In Hungary, the increase accelerated strongly after 25 years (note that under the alternative definition of onset of postponement, in Hungary it would have started 13 years later and thus Hungary would have been clustered with the Czech Republic, Slovakia, and Estonia). Importantly, the increase in the share of women having children after age 40 is not levelling off. Only in Sweden and Denmark has the increase become somewhat less pronounced. This is also the case for second births (online Supporting Information), but more years of observation are necessary to draw any conclusions.

## Late fertility among women and men

**FIGURE 4:**
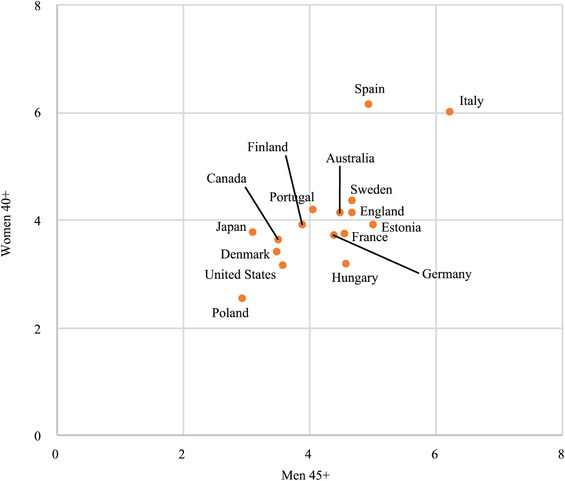
Contribution of women aged 40+ years to the TFR versus contribution of men aged 45+, 2014 SOURCE: Human Fertility Database for females; ABS, Australia; Eurostat, Denmark; Canada, 2011; France and Germany 2013. Dudel and Klüsener (2019) for males (in Human Fertility Collection).

The age threshold of “late” childbearing is earlier for women than for men. In fact, across all countries the contribution of births at age 40+ among women is very similar to the contributions at age 45+ for men (between 2 and 6 percent), and there are no outliers (Figure 4). With quite similar age differences between partners across low‐fertility countries today (Nitsche et al. 2018), the mechanisms of family formation certainly contribute to the regularity of these gender differences in late fertility. Besides the five‐year age difference, male late fertility is spread much more widely across late ages than that of women, continuing up to age 59 and beyond. Male fertility at age 55 and over still accounted for up to 0.5 percent of the TFR in some countries in 1990 and 2014, a proportion higher than that of births to women aged 45 and over (Table 2). Only a few thousand women give birth after age 50 across Europe and the United States (Sobotka and Beaujouan 2018).

**TABLE 2:** Contribution of late births to TFR and increase between 1990 and 2014, men and women

	Women			Men					
	45+			45+			55+		
Country	1990	2014	Percentage increase	1990	2014	Percentage increase	1990	2014	Percentage increase
Australia	0.1	0.3	419	3.1	4.7	49	0.5	0.5	16
Canada*	0.0	0.2	423	2.5	3.5	40	0.3	0.3	11
Denmark	0.0	0.2	272	2.5	3.5	39	0.3	0.3	4
England	0.1	0.3	198	3.1	4.7	53	0.5	0.5	0
Estonia	0.1	0.2	210	2.1	5.0	142	0.2	0.4	142
Finland	0.1	0.2	144	2.5	3.9	57	0.2	0.3	29
Germany*	0.1	0.2	172	2.7	4.4	64	0.2	0.4	61
Hungary	0.0	0.1	257	1.4	4.6	222	0.2	0.4	191
Poland	0.1	0.1	51	2.0	2.9	44	0.2	0.2	30
Portugal	0.2	0.2	48	2.7	4.1	48	0.1	0.1	19
Spain	0.2	0.4	168	2.6	4.9	90	0.2	0.5	131
Sweden	0.1	0.2	340	2.2	4.5	101	0.0	0.0	−100
United States	0.1	0.2	292	2.8	3.6	29	0.4	0.4	−9

SOURCE: Human Fertility database for females; ABS, Australia; Eurostat, Denmark; Canada, 2011; France and Germany 2013. Dudel and Klüsener (2019) for males (in Human Fertility Collection).

The increase in late childbearing also concerns men, among whom births at age 45+ represented up to 3.1 percent of the TFR in 1990 and up to 5 percent in 2014 (Table 2). For women, these proportions were, respectively, 0.2 and 0.4 percent. In a few countries, the increase in contribution from age 45 onwards was equivalent for men and women (Hungary, Poland, Portugal), but in most countries it was strongest among women. Particularly in the English‐speaking countries (Australia, Canada, USA, and to a lesser extent England), the increase among women was around 10 times that of men. These results generally hold when comparing the rise in female contributions above age 40 and in male contributions above age 45. The increase in births at ages above 55 was also quite small among men in most countries, and even fell back in Sweden and the United States. With an initially lower incidence of very late fertility than men, it is generally women who have seen the largest increase in recent decades.

## Discussion and research avenues

This study confirms that the characteristics of late fertility have changed since the 1950s: while at that time most late births were higher order births, today they are primarily first and second births. In the past, cross‐country diversity in late fertility thus reflected differences in family size. Today, it mostly reflects the stage of fertility postponement that each country has reached. “Exposure” to late childbearing is an essential aspect of late fertility. For instance in the 1950s, first births at late ages among women occurred relatively more often than in the 1970s, and this was certainly linked to later marriage at that time. In addition, in times of natural conception and very large families, extremely late second births (48+) were more frequent than extremely late first births. Today, extremely late childbearing mostly concerns first births. Childless women who want at least one child are challenging the natural fertility barriers, while older mothers often appear to forego a second birth.

We observed that the rise in late childbearing is continuing and even accelerating in several countries. In terms of the contribution of births at age 40 and above to the female TFR, the levels of the 1950s have often not been surpassed. However, the contributions of late first births to first birth rates have risen to values not observed in the second half of the twentieth century, and certainly never before that. Today, late first births are also frequent in Central and Eastern Europe, and their prevalence across the low‐fertility countries depends largely on the time since onset of childbearing postponement in each country. Still, in countries where late first births were initially rare, the increase has been generally faster than in other countries of the same region; in the Czech Republic, Slovakia, and Estonia, it began almost immediately after the onset of postponement and was particularly fast. In Austria, postponement onset was rather late but the pace of increase in late first births was fast relative to the other Western countries.

In Italy, Spain, and Japan, late first births have risen to particularly high levels, in relative and absolute terms. These countries, with very low TFRs and high levels of childlessness, offer little institutional support to combine a career and parenting and, for many years, offered a rather traditional “marriage package” to women that was unable to satisfy their general aspirations to gender equality (De Rose and Racioppi 2008; Bumpass et al. 2009; Arpino, Esping‐Andersen, and Pessin 2015). Spain and Italy were also particularly affected by chronic youth unemployment and the Great Recession (Matysiak et al. 2018). Many women whose lives were difficult during the usual childbearing years may have tried to have at least one child “before it was too late” after repeated birth postponements. Their high levels of late fertility are somehow setting an ever‐later threshold for childbearing capacity in other countries where late fertility is growing quickly. These other countries still have a considerable margin for postponing childbearing toward “latest‐late” fertility, but probably at the cost of more recourse to ART and of more involuntary childlessness. In our study, we found no clear link between the markers of the SDT and late childbearing prevalence: In some countries with large rises in union dissolution and major normative changes, such as the Nordic countries, the relative share of late fertility is not particularly large, while in others where the SDT started later, such as Italy, prevalence is increasing at a remarkable pace.

The upturn in late fertility has spawned a growing number of studies that investigate its epidemiological, social, and demographic consequences. Very good overviews of the positive and negative individual consequences of delayed motherhood in today's society are available in the literature (Mills et al. 2011; Schmidt et al. 2012; Myrskylä, Barclay, and Goisis 2017). On the one hand, today's older parents have a better quality of life, providing their children with more economic resources (Powell, Steelman, and Carini 2007) and greater stability (Musick and Michelmore 2018); older mothers are less likely to smoke during pregnancy and more often have high socioeconomic status (Goisis, Schneider, and Myrskylä 2018a); they also experience higher subjective well‐being after the birth of a child (Myrskylä and Margolis 2014). On the other hand, childbearing from age 35 incurs more health risks for the mother and child (e.g., Schimmel et al. 2015), despite huge improvements in perinatal healthcare since the 1990s (Kotelchuck 2007; WHO 2014). Though it is generally acknowledged that lifestyle is more important than mother's age in explaining negative pregnancy outcomes (Myrskylä et al. 2017), old age at childbearing remains a risk factor.

Men's reproductive lifespan extends over many more years than women's, and only after age 55 does their contribution to the TFR become comparable to that of women aged 45 or more. Though more rarely mentioned, late fatherhood also has positive and negative consequences. The positive socioeconomic consequences for their children are by and large the same as those offered by older mothers (Powell et al. 2007). Research has also found negative consequences for risk of conception, pregnancy outcomes, and child health among men as early as age 40 (La Rochebrochard and Thonneau 2002; Nybo Andersen and Urhoj 2017). Socioeconomic characteristics only partially offset the risk of low birth weight and preterm delivery due to late paternal age (Goisis et al. 2018b). Finally, given that men can become fathers up to very late ages, and that their life expectancy is shorter than women's, a further risk is that of the child losing its father while still young. Indeed, based on simulations, Flammant (2019) suggests that fertility postponement may help to explain why the number of orphans in France has decreased so slowly in recent years. One of our findings moderates this factor, however: the increase in late fertility between 1990 and 2014 among men was much more limited than among women, and extremely late fatherhood has even declined in a few countries.

The stronger acceleration of late fertility among women than men also reflects the more radical change in women's situation in recent decades. Women's life trajectories are increasingly similar to those of men (Lesnard et al. 2016), and the massive increase in educational enrolment has concerned women more than men (Breen et al. 2010). In addition, studies in the Netherland and Spain show that since 1990 the age difference between married partners has tended to decrease at older ages, or after controlling for age (Van Poppel et al. 2001; Esteve, Cortina, and Cabré 2009), and this also suggests greater uniformity. So far, women are postponing more than men and becoming more similar to them in many respects. Nonetheless, in terms of fertility, women are likely to become increasingly constrained by age‐related infecundity, while men can continue to postpone fatherhood if necessary. Rather than tending toward convergence, gender inequalities in childbearing may therefore be reinforced. Comparison of the recent compositional change in characteristics of late mothers and fathers, notably in educational attainment and labor force participation, as well as assortative mating behaviors, would certainly bring additional insights on future trends.

Since the upturn in late motherhood in the 1980s–1990s, particularly for first and second births, the trend has often been viewed as a “social problem,” not just by the media but also by governments (Moguérou et al. 2011; Budds, Locke, and Burr 2013). Late fatherhood has always existed, and many mothers in the past had their fourth or fifth child at a late age without raising concerns of this type (Moguérou et al. 2011). Nonetheless, though Frank, Bianchi, and Campana (1994) state that “the existence of [assisted reproductive] technology is bound to modify thoughts and attitudes to motherhood” (p. 366), the tendency is still to make couples, but particularly women, individually responsible for the timing of their childbearing (Marshall and Woollett 2000). However, fertility postponement is a societal phenomenon that arises from the changing economic conditions and the transformation of life circumstances (Mills et al. 2011; Cooke Mills and Lavender 2012). It results from the choices women and couples have to make throughout their life course in transformed social settings, and more than ever before, those who wish to have children need support rather than blame for having them “too” late.

The current context of ongoing increase in late childbearing calls for reflection on ways to improve support for those who have reached later ages without being able to form a family. In some countries, this may involve a change in the legal and medical approach to ART (Vialle 2014). In addition, older pregnant women need close perinatal follow‐up, which has proved to be effective in decreasing adverse maternal and child outcomes (Till, Everetts, and Haas 2012; WHO 2015; AAP Committee on Fetus and Newborn and ACOG Committee on Obstetric Practice 2017; Marozio et al. 2017). Today more than ever, the growing demand for childbearing at advanced ages is creating a need for cutting‐edge medical assistance and considerate older mothers’ management to prevent pregnancy complications (Marshall and Woollett 2000; Carolan and Frankowska 2011).

## Research avenues

Further investigations are needed to assess empirically whether an age limit to childbearing, leading to a visible reduction in fertility levels, has been reached due to fertility postponement. In the mid‐2000s, Billari et al. (2007b) did not find strong evidence that first births are becoming increasingly compressed into a small (late) age range. Late fertility has increased substantially since then, and in countries where fertility postponement is most advanced, first birth dispersion has stabilized in the last decade (Nathan and Pardo 2019). Studies of “rectangularization” of fertility at older ages could be repeated, as well as empirical exploration of the link between fertility postponement and fertility and would probably yield more substantive results, particularly in countries such as Italy or Spain where the delay in entry into parenthood has been massive.

Knowledge of changes over time in fertility intentions at later ages would improve our understanding of the “demand” side of late childbearing. In Austria, fertility intentions at very late ages have risen, and the mismatch between cohort intentions to have children at later ages and late childbearing is increasing (Beaujouan 2018). These findings suggest that, despite the adaptation of women's fertility intentions to their life‐course experience, the increase over time in ultimate childlessness and the decrease in family size do not necessarily reflect women wishes when approaching the end of their reproductive life. On the other hand, except among those who were childless, couples in Brazil were in general very satisfied with their family size, even if it was smaller than initially intended (Alves de Carvalho et al. 2018). The way women and men adapt to fertility postponement and deal with their unfulfilled intentions to have children is important to explore.

Another avenue concerns the use of ART for late conceptions in different countries, an area of research so far held back by the limited availability and comparability of data (Calhaz‐Jorge et al. 2017). Laws associated with use of ART and its usual practice vary substantially from one country to another, sometimes restricting its use despite potentially strong demand, or resulting in cross‐border reproductive care (Ferraretti et al. 2010; Rozée Gomez and La Rochebrochard 2013; Vialle 2014; Präg and Mills 2017). Further research on the demand for ART and its actual use today among “late starters” or “late enlargers” is still needed.

## Supporting information

Supporting Information
